# Integrating Behavioral Health and Primary Care (IBH-PC) to improve patient-centered outcomes in adults with multiple chronic medical and behavioral health conditions: study protocol for a pragmatic cluster-randomized control trial

**DOI:** 10.1186/s13063-021-05133-8

**Published:** 2021-03-10

**Authors:** Abigail M. Crocker, Rodger Kessler, Constance van Eeghen, Levi N. Bonnell, Ryan E. Breshears, Peter Callas, Jessica Clifton, William Elder, Chet Fox, Sylvie Frisbie, Juvena Hitt, Jennifer Jewiss, Roger Kathol, Kelly Clark/Keefe, Jennifer O’Rourke-Lavoie, George S. Leibowitz, C. R. Macchi, Mark McGovern, Brenda Mollis, Daniel J. Mullin, Zsolt Nagykaldi, Lisa Watts Natkin, Wilson Pace, Richard G. Pinckney, Douglas Pomeroy, Alexander Pond, Rachel Postupack, Paula Reynolds, Gail L. Rose, Sarah Hudson Scholle, William J. Sieber, Terry Stancin, Kurt C. Stange, Kari A. Stephens, Kathryn Teng, Elizabeth Needham Waddell, Benjamin Littenberg

**Affiliations:** 1 University of Vermont, Burlington, VT USA; 2 Arizona State University, Tempe, AZ USA; 3 School of Medicine, University of Colorado, Aurora, CO USA; 4 Wellstar Health System, Marietta, GA USA; 5 University of Houston College of Medicine, Houston, TX USA; 6 University at Buffalo, Buffalo, NY USA; 7 University of Minnesota, Minneapolis, MN USA; 8 Stony Brook University, Stony Brook, NY USA; 9 School of Medicine, Stanford University, Palo Alto, CA USA; 10 University of Washington, Seattle, WA USA; 11 School of Medicine, University of Massachusetts, Worcester, MA USA; 12 Health Sciences Center, University of Oklahoma, Oklahoma City, OK USA; 13 DARTNet Institute, Aurora, CO USA; 14 CHE Behavioral Health Services, New York City, NY USA; 15 National Committee for Quality Assurance, Washington, DC, USA; 16 University of California San Diego, San Diego, CA USA; 17 MetroHealth System, Cleveland, OH USA; 18 Case Western Reserve University, Cleveland, OH USA; 19 Oregon Health & Science University, Portland, OR USA

**Keywords:** Behavioral health, Primary care, Multiple chronic conditions, Pragmatic trials, Randomized control trial

## Abstract

**Background:**

Chronic diseases that drive morbidity, mortality, and health care costs are largely influenced by human behavior. Behavioral health conditions such as anxiety, depression, and substance use disorders can often be effectively managed. The majority of patients in need of behavioral health care are seen in primary care, which often has difficulty responding. Some primary care practices are providing integrated behavioral health care (IBH), where primary care and behavioral health providers work together, in one location, using a team-based approach. Research suggests there may be an association between IBH and improved patient outcomes. However, it is often difficult for practices to achieve high levels of integration. The Integrating Behavioral Health and Primary Care study responds to this need by testing the effectiveness of a comprehensive practice-level intervention designed to improve outcomes in patients with multiple chronic medical and behavioral health conditions by increasing the practice’s degree of behavioral health integration.

**Methods:**

Forty-five primary care practices, with existing onsite behavioral health care, will be recruited for this study. Forty-three practices will be randomized to the intervention or usual care arm, while 2 practices will be considered “Vanguard” (pilot) practices for developing the intervention. The intervention is a 24-month supported practice change process including an online curriculum, a practice redesign and implementation workbook, remote quality improvement coaching services, and an online learning community. Each practice’s degree of behavioral health integration will be measured using the Practice Integration Profile. Approximately 75 patients with both chronic medical and behavioral health conditions from each practice will be asked to complete a series of surveys to measure patient-centered outcomes. Change in practice degree of behavioral health integration and patient-centered outcomes will be compared between the two groups. Practice-level case studies will be conducted to better understand the contextual factors influencing integration.

**Discussion:**

As primary care practices are encouraged to provide IBH services, evidence-based interventions to increase practice integration will be needed. This study will demonstrate the effectiveness of one such intervention in a pragmatic, real-world setting.

**Trial registration:**

ClinicalTrials.gov NCT02868983. Registered on August 16, 2016.

## Administrative information

**Table:** 

Title{1}	Integrating Behavioral Health and Primary Care (IBH-PC) to improve patient-centered outcomes in adults with multiple chronic medical and behavioral health conditions: study protocol for a pragmatic cluster-randomized control trial.
Trial registration {2a and 2b}.	ClinicalTrials.gov identifier: NCT02868983
Protocol version {3}	April 15, 2019. Version 4.
Funding {4}	Patient-Centered Outcomes Research Institute (PCORI) Award PCS-1409-24372
Author details {5a}	^1^University of Vermont ^2^Arizona State University ^3^University of Colorado School of Medicine ^4^Wellstar Health System ^5^University of Houston College of Medicine ^6^University at Buffalo ^7^University of Minnesota ^8^Stony Brook University ^9^Stanford University School of Medicine ^10^University of Washington ^11^University of Massachusetts Medical School ^12^University of Oklahoma Health Sciences Center ^13^DARTNet Institute ^14^CHE Behavioral Health Services ^15^National Committee for Quality Assurance ^16^University of California San Diego ^17^MetroHealth System ^18^Case Western Reserve University ^19^Oregon Health & Science University
Name and contact information for the trial sponsor {5b}	Patient-Centered Outcomes Research Institute (PCORI) 1828 L Street NW, Suite 900 Washington, CD 20036 Phone: (202) 827-7700; Fax: (202) 355-9558; Email: info@pcori.org
Role of sponsor and funder {5c}	The study funder (PCORI) has no role in the design, execution, analyses, interpretation of data, or decision to submit results for this study.

## Introduction

### Background and rationale {6a}

The chronic diseases that drive the majority of morbidity, mortality, and health care costs in America and around the globe are largely influenced by human behavior. Tobacco use, poor diet, alcohol and substance use disorders, and physical inactivity together account for 38% of all deaths in the USA [1]. Insomnia, anxiety, depression, stress, and non-adherence to recommended treatment contribute additional morbidity and mortality. The presence of both chronic medical and behavioral health conditions drive poorer outcomes and higher costs than either alone [2, 3].

Behavioral Health Care (BH) is a short-hand term representing a wide variety of services that have often been physically, operationally, and even ideologically isolated from one another. The working definition of BH for this study (modified from Baird, 2014 [4]) is mental health care, substance use disorder care, health behavior change, and attention to family and other psychosocial factors that affect a person or a family unit. Conditions addressed as part of BH include depression, anxiety, stress, insomnia, overeating, inactivity, smoking, medication non-adherence, chronic pain, problem drinking, substance use disorder, and family distress.

Behavioral health conditions can often be effectively managed, resulting in improved outcomes for patients, their families and the health care system. Several psychological and behavioral techniques, including Cognitive Behavioral Therapy [5–8] and the IMPACT collaborative care model [9], improve outcomes, best demonstrated for depression.

The majority of patients in need of BH are seen in primary care, but that setting has difficulty responding to the full set of needs. Forty percent of primary care patients have BH needs and the need is even higher among those with chronic medical conditions [10]. Although some primary care providers (PCPs) are skilled and effective at delivering BH [11], many do not have the training, time, or inclination [12]. High-quality BH is the single most difficult area of medical care for patients to access [13] and nearly 70% of the more serious behavioral health conditions in primary care are neither assessed nor treated [14].

The traditional approach to delivering BH in much of the world is for PCPs to refer patients to specialists (psychiatrists, psychologists, counselors, and therapists) outside the primary care setting. Unfortunately, high-quality services are often not available, the referral process is challenging, and treatment initiation rates are low, in part due to the perceived stigma of seeing a mental health specialist [15–17]. Communication between mental health specialists and PCPs is often hampered by confidentiality concerns, time constraints, legal restraints, transportation barriers, and traditional practice habits. Fifty percent to 90% of primary care referrals made to out-of-office mental health practitioners fail to result in an appointment [18]. Of those patients who do schedule an appointment, most never initiate care [19]. When care is initiated, most of that care is not evidence-supported [20].

In response to these barriers, many primary care practices have instituted co-located services in which a Behavioral Health Provider (BHP) such as a psychologist or counselor and PCP deliver care in the same location [21]. This model eliminates some of the barriers to access, but the BHP often operates independently from the PCPs with separate hours, space, appointment systems, and medical records. Often, there is little communication or shared records between the two disciplines. There is little evaluation of such interventions [22].

An alternative approach to delivering BH for primary care patients is Integrated Behavioral Health Care (IBH) in which the BHP is a full member of the practice who shares workspace, infrastructure, records and support systems, participates fully in the life of the practice, and collaborates closely with PCPs in patient assessment and management. Beyond co-location, IBH includes support for population management, protocol-driven evidence-supported care, guideline-based external referral, use of brief visits, systematic needs identification, and practice-wide approaches to patient engagement.

The working definition of IBH (adapted from Korsen, 2013 [23]) for this study is the care a patient receives when PCPs and BHPs use a collaborative system of care, rather than focusing on separate approaches. Its goals are better and more efficient care of mental health conditions, substance use disorders, and chronic medical illnesses; better recognition and management of life stressors, crises, and stress-related physical symptoms; improved health behaviors; and more thoughtful and efficient health care utilization. Research suggests there may be an association between IBH and patient outcomes [24].

For primary care practices to effectively shift from co-located services to IBH, a supported practice change process is required [4]. While IBH checklists identifying integration elements exist [23, 25], a comprehensive intervention that systematically combines practice redesign with quality improvement (QI) coaching, provider and staff education, and collaborative learning does not. The Integrating Behavioral Health and Primary Care study (IBH-PC) responds to this need by testing the effectiveness of a comprehensive practice-level intervention (the IBH-PC toolkit) designed to improve outcomes in patients with multiple chronic medical and behavioral health conditions by increasing a practice’s degree of behavioral health integration. We sought to deploy the toolkit in a broad array of primary care practices and test its impact on patient functional status as well as the practices’ level of integration. We chose a pragmatic randomized trial design to allow our analysis and conclusions to be both rigorous and applicable to a broad range of practices.

### Objectives {7}

#### Hypothesis

Increased integration of evidence-supported behavioral health and primary care, compared to simple co-location of providers, will improve patient-centered outcomes in patients with multiple chronic medical and behavioral health conditions.

#### Research questions

1. Does using the IBH-PC toolkit, a practice-level intervention, affect patient-centered outcomes in adults with multiple chronic medical and behavioral health conditions?
2. Does using the IBH-PC toolkit affect the degree of practice-level behavioral health care integration in primary care practices?
3. What factors support or impede successful integration of behavioral health care into primary care practices?
4. What are the costs of implementing the IBH-PC toolkit?

### Trial design {8}

The IBH-PC study is a two-arm, parallel, superiority, pragmatic, cluster-randomized trial that treats patients as the units of analysis clustered within practices that are the units of intervention. Practices are assigned in a 1:1 allocation ratio.

## Methods: Participants, interventions, and outcomes

### Study setting {9}

**Fig. 1:**
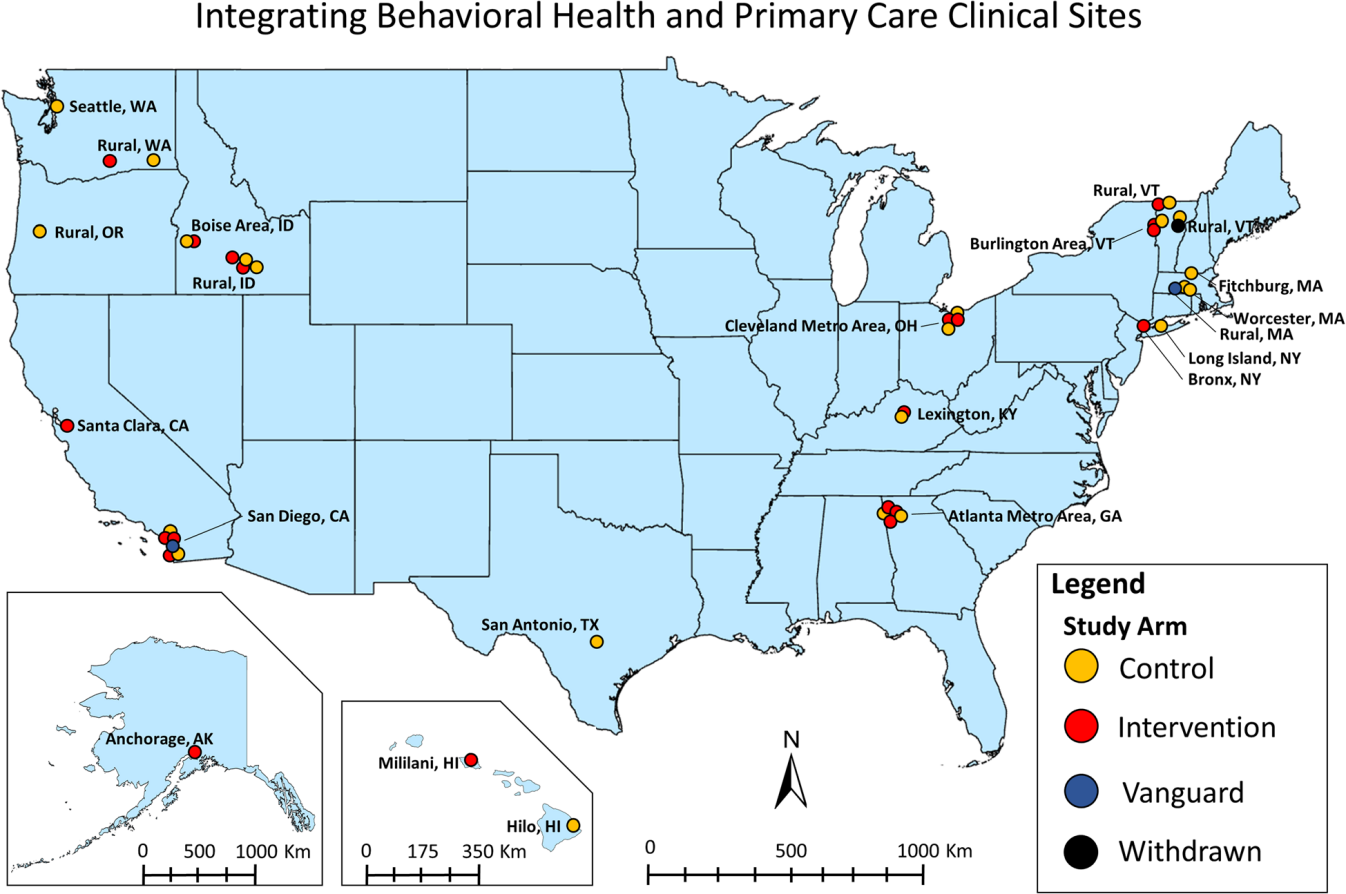
Integrating Behavioral Health and Primary Care (IBH-PC) clinical sites

The IBH-PC study takes place in 45 primary care practices across the United States (Fig. 1). Practices represent a diverse distribution of geographic regions, population densities (urban vs. rural), patient population size, specialty (family vs. internal medicine), community health centers, federally qualified health centers, nonprofit and for-profit organizations, resident training sites, and ownership (hospital or health system, academic, private).

### Eligibility criteria {10}

#### Study practices

Primary care practices are eligible to participate if they:

- Have at least one PCP and at least one BHP onsite (co-located),
- Have at least 0.5 full-time equivalent BHPs licensed to practice independently,
- Commit to maintaining onsite BHP for the duration of the study,
- Provide the research team with access to electronic medical records (EMRs) to identify patients with specific medical and behavioral health conditions for recruitment,
- Are willing to complete survey instruments periodically throughout the study, and
- Are willing to be randomized to either the active or control arm.

For practices with more than 10% of their revenue generated by Medicare, at least one BHP per practice must be eligible to bill Medicare. Practices are ineligible if they are already undergoing, or plan to undergo, another QI initiative similar to the IBH-PC intervention or if they already have an advanced level of BH integration as evidenced by a total Practice Integration Profile score (PIP) above 75. The PIP is a survey of 30 items completed by primary care providers and staff about their own practice used to measure practice level integration [26, 27].

### Study patient participants

Patients are eligible to participate if they:

- Are at least 18 years old,
- Are an active patient of a participating study practice as evidenced by at least two visits in a period of 24 months for any purpose, including at least one in the most recent 6 months,
- Are willing to complete three surveys over 2 years, and
- Have both an eligible chronic medical condition and an eligible chronic behavioral health condition, or at least three eligible chronic medical conditions. Eligible chronic medical conditions include arthritis; obstructive lung disease including emphysema, chronic bronchitis, or asthma; non-gestational diabetes; and heart disease manifested as heart failure or hypertension. Eligible behavioral health conditions include mood disorder (anxiety or depression), chronic pain (including headache, migraine, neuralgia, fibromyalgia, or chronic musculoskeletal pain), insomnia, irritable bowel syndrome, and substance misuse (substance use disorder, tobacco use, or problem drinking).

To determine patient eligibility, medical records are reviewed for a period of 24 months with the most recent date being within 12 months of the study start at each practice. Within this period, evidence of age, minimum number of visits, and medical and behavioral health conditions will be determined.

Evidence of medical conditions may take the form of a specific diagnosis on a problem list. Evidence of diabetes may also be indicated by 3 months of insulin or other diabetes medications (excepting metformin which is not specific to diabetes) or any hemoglobin A1C value greater than 6.4%. Evidence of heart disease may also be indicated by 3 months of cardiac medications specific to hypertension or heart failure (excepting beta-blockers and other medications with broader indications), or 3 sequential blood pressure measurements with mean systolic pressure > 140 mmHg or mean diastolic pressure > 95 mmHg.

Evidence of a behavioral health condition may take the form of a specific diagnosis on a patient’s problem list, 3 months of certain medications used for behavioral conditions (antidepressants, anxiolytics, opioids, antineuropathic agents, agents for alcohol use disorder or smoking cessation, etc.), or persistent failure to attain physiologic control of a medical problem evidenced as systolic blood pressure > 165 mmHg for 3 months or more or A1C > 9% for 6 months or more.

#### Who will take informed consent? {26a}

Eligible patient participants are identified by review of EMRs in each participating primary care practice [28]. Acting as trusted third party, the DARTNet Institute [29] reviews the EMR data, identifies potential patient participants, and offers participation.

Eligible patients are mailed a letter describing the purpose of the study and that, if they choose to participate, they are requested to complete a brief set of surveys at three timepoints via web, paper, or phone. The letter also describes the process to opt-out as well as provides a phone number to call for more information on the study. The DARTNet Institute follows-up the letters with phone calls to eligible patients. Consent is administered and documented by phone, paper, or via web at the completion of the baseline set of surveys. Participating patients provide consent to link their EMRs to survey data and may discontinue participation at any time. All study materials are available in both Spanish and English.

#### Additional consent provisions for collection and use of participant data and biological specimens {26b}

The study consent process includes permission for additional analyses of collected data. No biological specimens are collected.

### Interventions

#### Explanation of the choice of comparators {6b}

The active comparator (the intervention) is the use of the IBH-PC toolkit to support a practice-level change process. Use of the IBH-PC toolkit is intended to increase the delivery of highly integrated behavioral health and primary care, which is hypothesized to result in improved patient outcomes. The control comparator is co-location of a behavioral health provider within or adjacent to the primary care practice (at the same street address), without additional integration [12]. Co-location was chosen as the control comparator as it is highly prevalent in the field and has emerged as a standard of care in this domain [10].

#### Intervention description {11a}

The intervention, the IBH-PC toolkit, is a set of implementation strategies consisting of four components:

1. An online educational curriculum,
2. A structured, team-based, practice redesign and implementation workbook,
3. Remote QI coaching services for internal QI facilitators, and
4. An online learning community.

The purpose of the IBH-PC toolkit is to guide and support primary care practices through a practice change process as they work to increase their degree of behavioral health integration through, for example, improvement of screening, case identification, management, or follow-up. Progress through the toolkit and use of its components are expected to be variable among participating practices, taking from 9 to 24 months.

##### Online education curriculum

The IBH-PC toolkit includes an asynchronous online curriculum on evidence-based concepts of IBH and methods of applying them. The curriculum includes one interprofessional course for all practice member roles and seven individual courses, targeting different practice member roles (practice manager, BHP, PCP, nurse, staff, IBH-PC facilitator) Courses take approximately 4–14 h to complete, depending on the practice member role. The online curriculum is housed at the Arizona State University and is made available through an online learning management platform [30]. It is intended, but not required, that all practice members complete the courses appropriate for their roles.

##### Practice redesign and implementation workbook

The redesign and implementation process is available as a workbook format (.pdf format) that is posted in an online, shared workspace. Practices are encouraged to establish interdisciplinary project teams, with a practice member serving as project leader and an on-site, internal facilitator. The workbook directs the project team through four stages: study start up and leadership engagement, planning the scope and boundaries of workflow redesign using a Lean Management approach [31–33], redesigning workflow with recommended tactics, and implementing those changes in the practice. It is intended, but not required, that the project teams complete all stages sequentially.

##### Remote quality improvement coaching

To support the project teams through the redesign process, external QI coaches work with individual practices. Each study practice is assigned a team of two coaches who meet with them via phone or web-meeting throughout the practice redesign process (up to 24 months). The remote QI coaches are part of the research team based at the University of Vermont (UVM). It is intended, but not required, that all project teams meet with their coaches on a regular basis.

##### Online learning community

The IBH-PC toolkit includes an online forum and learning community created using an open source bulletin board system [34]. It provides a platform for practice members and other IBH-PC stakeholders to communicate with one another. Discussion topics vary and are primarily focused on practice member needs and interests. It is intended, but not required, that all project teams engage in the online learning community through regular participation by at least one team member.

#### Criteria for discontinuing or modifying allocated interventions {11b}

Study practices randomly assigned to the intervention are encouraged to use the IBH-PC toolkit completely and sequentially, as prescribed. However, practices may use the toolkit out of sequence, modify sections, or skip them completely. Given the low risk of this practice-level intervention, there are no early stopping rules.

#### Strategies to improve adherence to interventions {11c}

The IBH-PC toolkit is designed to encourage practice adherence to the intervention through engagement with a remote QI coaching team, access to continuing education credits as part of the online education curriculum, and creating a sense of community with standards of normal behavior through the online learning community.

#### Relevant concomitant care permitted or prohibited during the trial {11d}

Participating practices may not participate in other practice change projects similar to the IBH-PC toolkit during the trial. However, they may use QI and other strategies to improve any aspect of their practice they see fit, including improving BH services.

### Provisions for post-trial care {30}

Not applicable. The study represents a 24-month practice level-intervention, there is no need for post-trial care.

### Outcomes {12}

#### Research question 1: does using the IBH-PC toolkit, a practice-level intervention, affect patient-centered outcomes in adult patients with multiple chronic medical and behavioral health conditions?

##### Primary outcomes

- Change in mean patient Patient-Reported Outcomes Measurement Information System [35] (PROMIS-29) scores from baseline to follow-up for eight domains:
  - Physical function
  - Anxiety
  - Depression
  - Fatigue
  - Sleep disturbance
  - Social functioning
  - Pain intensity
  - Pain interference

##### Secondary outcomes

- Change in other mean patient-reported measures of health and quality of care from baseline to follow-up.
  - Empathy [36]
  - Medication adherence [37]
  - Healthcare utilization [38]
  - Time lost due to disability [39]
  - Physical function [40]
  - Patient centeredness
- Change in mean measures of patient disease control from baseline to follow-up for specific subgroups identified from EMRs.
  - Depression [41]
  - Anxiety [42]
  - Asthma symptoms [43, 44]
  - Substance use disorder [45]
  - Problem drinking [46]

#### Research question 2: does using the IBH-PC toolkit affect the degree of practice-level behavioral health care integration in primary care practices?

##### Secondary outcomes

- Change in median PIP total score from baseline to follow-up
- Change in median PIP domain scores from baseline to follow-up
  - Practice Workflow
  - Clinical Services
  - Integration Methods
  - Case Identification
  - Patient Engagement
  - Workspace Arrangement and Infrastructure

#### Research question 3: what factors support or impede successful integration of behavioral health care into primary care practices?

##### Secondary outcomes

- Identification of practice characteristics and external factors supporting or impeding integration as measured by changes in PIP scores.

#### Research question 4: what are the costs of implementing the IBH-PC toolkit?

##### Secondary outcomes

- Total estimated costs of using the IBH-PC toolkit over a period of 24 months, excluding the costs and revenues associated with ongoing clinical operations.

##### Participant timeline {13}

**Fig. 2:**
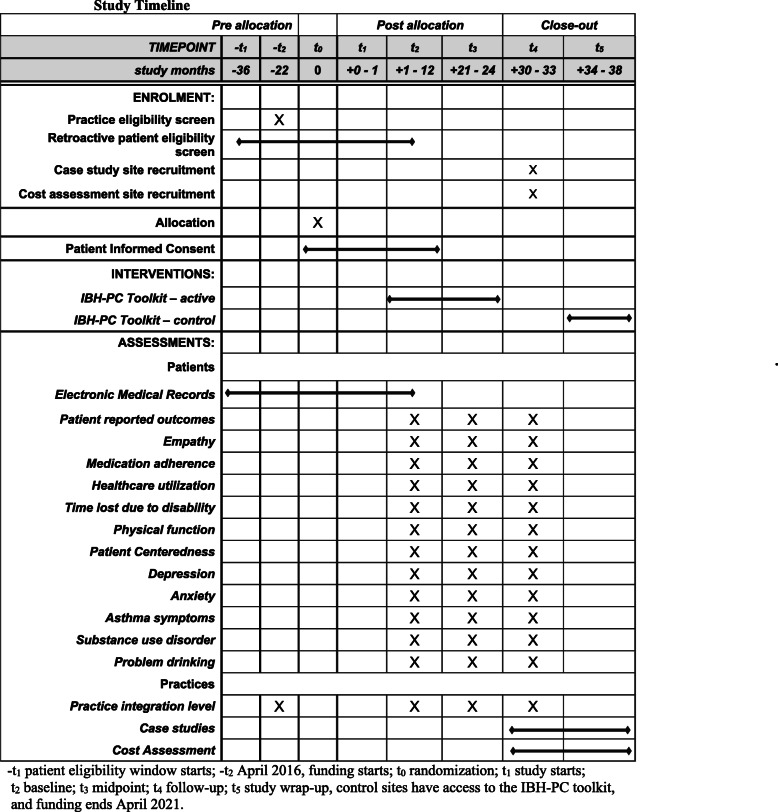
Study timeline

Practices are screened on a rolling basis for study eligibility and will therefore have varying randomization and study start dates. Moreover, it is anticipated that practices will move through the IBH-PC toolkit at different rates. Therefore, the timeframes represented in Fig. 2 are anticipated averages across all study practices.

### Sample size {14}

Sample size calculations were adjusted for the cluster randomized design [47]. To achieve a nominal significance level of 0.05 for the main hypothesis that any one or more of the eight PROMIS primary outcomes achieves significance, we will apply the Bonferroni correction [48] requiring *P* < 0.05/8 = 0.00625. All PROMIS domain scales are normalized to a standard deviation of 10 points [35]. We assume that the within-practice correlations of the PROMIS scales are 0.03 (similar to the SF-12 [49]). Although minimally important differences [50] are not fully specified, they range from 2 to 8 points for other instruments in the PROMIS series [51, 52].

Power calculations were conducted prior to study recruitment assuming 75 subjects from 40 practices, giving the study 3000 subjects and 90% power to detect a difference in any of the 8 PROMIS scales of 2.5 points or more. With 45 practices recruited, 43 practices randomized to intervention or control, and 2 practices considered as “Vanguard” the study recruitment meets these power and sample size expectations.

### Recruitment {15}

Recruitment of primary care practices to participate in the study leverages the professional networks of the study’s leadership and co-investigator teams. Study leadership calls potential practices, screening for interest and eligibility.

To recruit study patient participants, the DARTNet Institute identifies potential eligible patients using electronic medical record data from each practice. The data extract contains information on patient visits, medications, and diagnoses. These data retrospectively span at least 24 months and include dates within 12 months of the study start date. Eligible patients are contacted by mail and/or phone with an invitation to participate in the study.

**Fig. 3:**
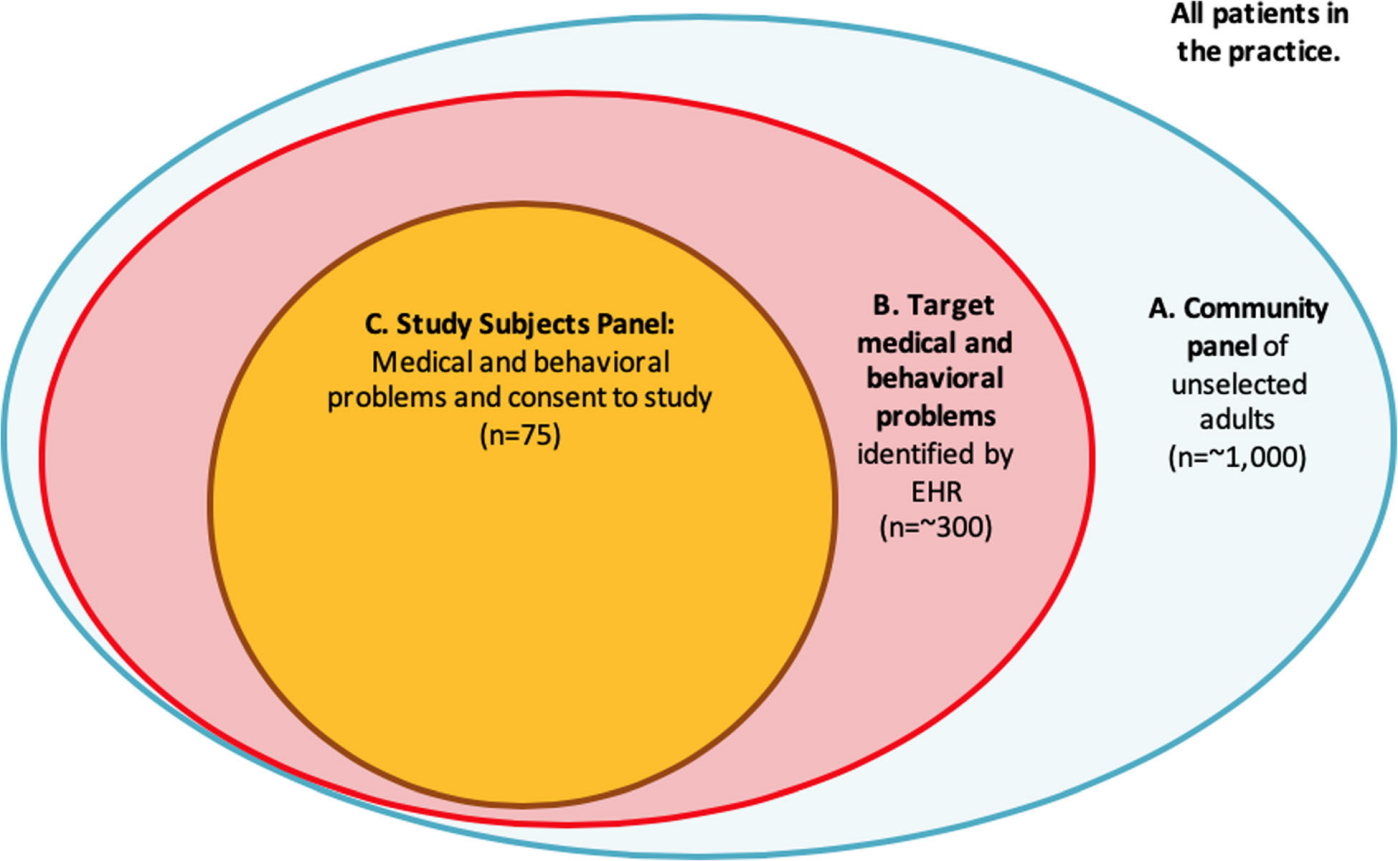
Patient participants identification schematic

We cannot sample from the entire practice panel of potentially eligible patients because, in most cases, the practices have many more patients with multiple chronic medical and behavioral health conditions than their BHPs can reasonably support, which would limit our ability to capture the effects of each practice’s case finding and clinical management changes on study outcomes. Nor can we allow each practice to know exactly who the consented patient participants are because the practice may tend to concentrate behavioral health resources on those patients in a way not usual in real-world practices, introducing bias. Therefore, we developed a “Community Panel” for each practice consisting of a random subset of the entire practice’s patient population of active adult patients (group A in Fig. 3). Within the Community Panel, some patients will have the target medical and behavioral conditions and be eligible for to participate in the study (group B). The study subjects (group C) are recruited from group B. Because practice change efforts to integrate behavioral health care usually involve increasing the workload of BHPs, the Community Panel size is proportional to the number of BHPs in the practice, approximately 1000 adults per 1.0 full-time equivalent BHP. The Community Panel allows us to conduct a pragmatic trial within the staffing constraints imposed by real-world conditions.

All practices receive the names of the patients who comprise their Community Panel during the baseline period of the study, but they are not told which ones are eligible for the study or which consented to participate. Control practices may choose to use the Community Panel in the course of their usual care. Active practices are encouraged to focus their redesign efforts (pertaining to screening, case identification, management, follow-up, etc.) on the patients in the Community Panel.

Approximately 8 participating practices will be purposefully selected for inclusion in a qualitatively-driven collective case study of factors that support or impede successful integration of behavioral health and primary care (research question 3). We will identify tiers of “high change” and “low change” practices, where change is defined as the difference between baseline and midpoint median PIP total scores. Practices will be recruited on a rolling basis as midpoint PIP scores become available. Site selection will be informed by emerging findings from both the qualitative data and successive phases of quantitative data that are slated to continue throughout the project period [53].

To estimate the costs of using the IBH-PC toolkit (research question 4), approximately 8 active practices will be recruited using a combination of purposeful and convenience sampling. Selection will be based on practice type, size, location, and capacity to participate in data collection activities. Recruitment and data collection will begin while practices are actively engaged with the IBH-PC toolkit (these sites need not be those selected for the qualitative analysis of research question 3.) The cost analysis was limited to 8 representative practices, in order to minimize data collection burden and optimize response.

### Assignment of interventions: allocation

#### Sequence generation {16a}

Eligible practices are assigned to the active or control arm using a stratified, randomized, approach, in blocks of 4. Randomization blocks are developed using R [54]. Practices are stratified based on degree of behavioral health integration at baseline (total PIP score < 50th percentile vs. total PIP score > =50th percentile) and geographical area.

#### Concealment mechanism {16b}

Randomization cards are a dark color, in order to prevent transparency, and individually sealed in opaque manila envelopes. Randomization envelopes are shuffled within block, numbered sequentially within strata and stored in a locked file cabinet. Potential subversion is guarded against by storing the randomization envelopes in a locked file cabinet and only accessing the envelopes when at least two members of the study team are present.

#### Implementation {16c}

When a practice is deemed eligible and ready to be randomized, a data analyst determines which stratum they belong to and opens the next randomization envelope within that stratum. She writes the practice’s name and date of randomization on the card, and then initials the card before filing it. The site’s randomization status is communicated to the Principal Investigator (PI) who sends an email to the practice and notifies the study Practice Change team.

### Assignment of interventions: blinding

#### Who will be blinded {17a}

Once a study practice has been randomized, their assigned study arm is not blinded. Practices are blinded as to who the patient participants are within their practice.

#### Procedure for unblinding if needed {17b}

Not applicable. This study is an unblinded, practice-level intervention.

### Data collection and management

#### Plans for assessment and collection of outcomes {18a}

The main source of outcomes data and descriptors are the patients and practices themselves. On-line surveys are collected and managed using the Research Electronic Data Capture (REDCap) system, a secure, web-based application designed to support data capture for research studies [55]. We also offer patients the option of completing the forms by telephone with a trained interviewer using a scripted interview, or via paper using a mail-in option. Outcome measures are collected at three timepoints: baseline, midpoint, and follow-up. The baseline period reflects the first 0–12 months of participation after randomization, midpoint is 9–12 months after baseline, and follow-up is 18–21 months after baseline. The Data and Analysis Team reviews all records for potential errors.

##### Study instruments

Research question 1: does using the IBH-PC toolkit, a practice-level intervention, affect patient-centered outcomes in adults with multiple chronic medical and behavioral health conditions?

Our primary outcome, the PROMIS-29 [35], is a set of person-centered measures of physical, mental, emotional, and social health included in the National Institute of Health’s (NIH) measurement toolbox. Rather than a disease-focused measurement system, it provides valid measures of Physical Function, Anxiety, Depression, Fatigue, Sleep Disturbance, Social Functioning, and Pain. These aspects of functioning and well-being are relevant across many chronic conditions and broadly represent “health-related quality of life.”

Additional instruments will be used to collect secondary outcomes:

- The Consultation and Relational Empathy [36] (CARE) survey is a 10-item validated self-report measure that assesses patients’ perception of provider empathy. The CARE has been shown to have excellent reliability (Cronbach’s alpha 0.92).
- The Modified Self-reported Medication-taking Scale [37] is the Morisky Green Levine Scale (MGLS) four-item self-report measure assessing adherence to prescription medication. The MGLS has been shown to have adequate reliability (Cronbach alpha = 0.61 [56]).
- The Utilization Patient Report [38] is a 3-item self-report measure asking patients to recall their healthcare utilization in the past year. Specifically, patients are asked to report utilization of visits to the emergency room, overnights in the hospital, and outpatient appointments to a healthcare provider.
- Time loss due to disability will be measured using the Restricted Activity Days survey [57]. This survey asks patients to report on the restriction of their daily lives due to illness and disability.
- Physical function will be measured using the Duke Activity Status Index [40] (DASI), a 12-item validated self-report measurement used to assess functional capacity. The DASI has been shown to have good to excellent reliability in several studies (Cronbach’s alpha ranging from 0.80 to 0.93 [58]) and correlates with peak oxygen uptake measured by exercise stress test.
- Patient-centered primary care will be measured using the Patient Centered Index (PCI), a 16-item questionnaire. This tool was developed for use in the IBH-PC study by a team of researchers, patients, and other stakeholders to assess how a patient perceives their care. Reliability and validation measures are not yet available.
- The 7-item Generalized Anxiety Disorder Scale [42] (GAD-7) is a self-report questionnaire developed and validated in a larger primary care patient sample to assess anxiety symptom severity. The GAD-7 has been shown to have excellent reliability in several studies (Cronbach’s alpha ranging from 0.79 to 0.91 [59]).
- The 9-item Patient Health Questionnaire [41] (PHQ-9) is a validated self-report measure of depression symptom severity. The PHQ-9 has shown to have good reliability (Cronbach’s alpha ranging from 0.86 to 0.89 [41]). Recognizing the sensitive nature of the questions on the PHQ-9 concerning suicidal thoughts and ideation, information on how to access the national suicide helpline was distributed with all paper study materials and was programmed into the electronic survey data capture system.
- The Asthma Symptom Utility Index [43, 44] (ASUI) is a 10-item validated self-report measure assessing control and quality of life as it relates to asthma symptoms. Reliability for the ASUI is considered good with a Cronbach’s alpha of 0.74 [43].
- Alcohol use will be assessed using 2-items from the Self-Report Habit Index-Alcohol [46] (SRHI-A). The SRHI-A has shown to have excellent reliability (Cronbach’s alpha 0.94 [46]).
- Substance Use Disorder will be assessed using the 5-item Substance Disorders subscale of the Global Assessment of Individual Needs-Short Screener [45] (GAIN-SS), a biopsychosocial screener for individuals presenting with substance use and mental health concerns. The GAIN-SS Substance Use subscale has been shown to have acceptable reliability (Cronbach’s alpha ranging from 0.77 to 0.84 [60]).

Research question 2: does using the IBH-PC toolkit affect the degree of practice-level behavioral health care integration in primary care practices?

The Practice Integration Profile (PIP) is a survey of 30 items completed by primary care providers and staff about their own practice [26, 27]. It provides a total integration score and 6 domain scores: practice workflow, clinical services, integration methods, case identification, patient engagement, and workspace arrangement and infrastructure.

Research question 3: what factors support or impede successful integration of behavioral health care into primary care practices?

A cross-case comparative analysis [53, 61, 62] will be conducted in selected practices. Researchers will collect data contained in field notes, such as observations of the physical layout of the practice, workflow, and practice meetings; transcripts of semi-structured interviews with coaches, cluster leaders, and practice staff; and relevant site documents.

Research question 4: what are the costs of implementing the IBH-PC toolkit?

A dedicated staff member from each of selected active study sites will periodically complete an in-depth program implementation Cost Assessment Tool (CAT). The CAT is a role-based time-effort evaluation instrument listing the number and type of practice staff participating in each toolkit activity. We will total the time various practice members engage with each component of the IBH-PC toolkit over a 24-month period and calculate the time-effort and cost in US dollars using median total compensation rates appropriate for the practice’s ZIP code and the practice staff’s professional roles. We will also include any supply, equipment, capital, or other non-personnel costs, including training time and coaching time associated with toolkit implementation. Time-effort data collected from participating practices by each participant and subtask will be analyzed for completeness, range and “face validity” of time estimates, and appropriateness of data to determine econometric outcomes. Key variables will then be estimated from global and sub-group averages, including total time and corresponding cost of program implementation steps, time/cost of the planning phase, and those of all implementation phases.

#### Plans to promote participant retention and complete follow-up {18b}

First, each practice receives financial support for a clinical leader and a practice manager to attend to data collection and other non-clinical (research-related) aspects of the project. Second, practice sites are organized into clusters of about 5–7 practices. Each cluster has a designated local Cluster PI (a behavioral or medical provider or researcher). The Cluster PI serves as a member of the research team and coordinates research activities, engages upper practice management, and provides liaison with the investigators. Third, all control practices have access to the intervention (the IBH-PC toolkit) upon completion of the follow-up assessments.

To encourage patient participants to remain engaged and complete the baseline and follow-up surveys, we include a midpoint survey data collection time point to minimize the time in between research contacts and pay patient participants $30 upon completion of each survey ($90 total).

### Data management {19}

The research team developed a written data governance policy to guide appropriate storage and use of study data. Data from consented patient participants are collected electronically, on paper, and over the phone. To complete baseline, midpoint, and follow-up patient surveys, the study team contacts patient participants by each of these three methods over a 3-month period until a response is received or the 3-month window is reached. The following steps are taken:

- All patient participants with email addresses are emailed at week zero.
- Three email reminders are sent every other day if there is no response.
- At week one, a paper survey is sent to the patient participant’s mailing address.
- At week five, a second batch of emails is sent followed by two phone calls 1 week later. If the patient participant cannot be reached via phone, a voicemail is left.
- At week eight, a second paper mailer is sent to patient participants who have yet to respond.
- At weeks nine through twelve, two more emails and two more phone calls are attempted before concluding efforts.

Data collected electronically via REDCap are automatically captured and stored in REDCap. Trained research assistants sort and enter paper surveys into REDCap and then they are filed and stored in a locked filing cabinet inside a locked office. Postal address, email, and phone number are verified for accuracy and updated as need. Phone surveys are conducted by trained research assistants and information is entered directly into REDCap. Patient participants who submitted surveys with missing data are contacted by telephone to complete the survey. Double data entry is performed, by a team of trained research assistants, on a randomly selected subset of 10% of the surveys received for each time point (baseline, midpoint, and follow-up). If the data entry error rate is greater than 0.05%, the entire batch of surveys is re-entered.

### Confidentiality {27}

Data from EMRs used to recruit patient subjects are extracted, transformed, loaded and curated from clinical practice sites by the DARTNet Institute. DARTNet uses a HIPAA-compliant sFTP cloud transfer system. This includes encryption of all data in transit and deletion of the data as soon as the transfer is complete. Data files transferred to DARTNet are immediately moved behind a second firewall where access is internet protocol address (IP)-to-IP controlled. All file manipulations occur behind this second firewall. Once DARTNet has completed subject recruitment, they use a similar system, Egnyte [63], to transfer the list of patients on a practice’s Community Panel to a practice.

Patient names, medical record numbers, and other unique identifiers are stripped and replaced with a unique study identifier before transferring EMR files to the UVM research team. Electronic medical record files contain data on healthcare visits, clinical measures including blood pressure and hemoglobin A1C, as well as medication and problem lists. Only research personnel authorized to access data for the proposed project have access to the data.

After transfer of EMR data to UVM, all source material, copies, data files, transcripts, and other research materials are maintained in a secure environment with access restricted to members of the study Data and Analysis Team.

Online patient and practice surveys are collected and managed using the REDCap system [55], a secure, web-based application designed to support data capture for research studies.

Physical copies of any patient surveys or other research documents with potentially identifying information are kept under lock and key. Digital materials are secured behind password-protected firewalls.

### Plans for collection, laboratory evaluation, and storage of biological specimens for genetic or molecular analysis in this trial/future use {33}

Not applicable. Biological specimens are not collected as part of this study.

### Statistical methods

#### Statistical methods for primary and secondary outcomes {20a}

Research question 1: does using the IBH-PC toolkit, a practice-level intervention, affect patient-centered outcomes in adults with multiple chronic medical and behavioral health conditions?

For the primary outcomes, we will use generalized linear mixed (GLM) models [64] of patient health status (PROMIS-29 scales) to perform intention-to-treat analyses [65] as a function of experimental condition (active vs. control) and patient characteristics, with patients clustered within practices. The parameters of interest are the central tendency (mean), statistical significance (*P* values), and 95% confidence intervals (CI) of the adjusted change in PROMIS-29 domain from baseline to follow-up. Each of the 8 outcome domains in the PROMIS-29 will be modeled individually as 8 separate hypotheses with adjustment for multiple comparisons and for clustering within practices.

Patient characteristics will be measured at baseline and will include age, gender, race, ethnicity, marital status, employment, education, and income.

For secondary patient outcomes, including change in other patient-reported measures of health and quality of care, and measures of patient disease control from baseline to follow-up, we will use similar GLM models as above but will not adjust for multiple comparisons.

#### Research question 2: does using the IBH-PC toolkit affect the degree of practice-level behavioral health care integration in primary care practices?

We will use GLM models of practice integration level (total PIP and PIP domains) to perform intention-to-treat analyses as a function of experimental condition (active vs. control) and practice characteristics. The parameters of interest are the central tendency (median), statistical significance (P values) and 95% CI of the change in total PIP and PIP domains from baseline to follow-up.

Practice characteristics will be measured at baseline and will include ownership type, specialty, training status, non-profit status, size, region, census-tract based variables, primary care medical home (PCMH) status, and % Medicare patients.

#### Research question 3: what factors support or impede successful integration of behavioral health care into primary care practices?

A cross-case comparative analysis [53, 61, 62] will be conducted for selected case study practices. Practices will be selected from the extremes of change in the main outcome variable, and additional practices maybe selected for confirming/disconfirming analyses [66]. Data collected from the individual case study sites will be analyzed to identify site-specific contextual factors that support or impede integration. Through multiple rounds of coding the data, researchers will organize and categorize related evidence on a topic from multiple data sources, assisting with pattern identification in preparation for thematic analysis [62, 67].

After analysis at the individual practice level, a cross-case study analysis will be conducted to identify prominent themes. This process allows for the in-depth examination of the individual practice sites to be followed by a cross-cutting analysis of integration processes. Findings will include themes and insights regarding the factors that supported or impeded successful integration of behavioral health care across the selected practices.

#### Research question 4: what are the costs of implementing the IBH-PC toolkit?

Descriptive statistical and basic econometrical (i.e., time/effort-cost estimation) methods will be used to summarize findings. Costs of IBH-PC toolkit implementation will be measured only, while research costs (identifying eligible patients, collecting and analyzing research data, etc.) and changes in operational costs and revenues *after* completion of the intervention will not be included.

### Interim analyses {21b}

Not applicable. Interim analyses and stopping guidelines are not planned for this study due to the low risk nature of the practice-level intervention.

### Methods for additional analyses (e.g., subgroup analyses) {20b}

#### Subgroup analyses

Subgroup analyses will be conducted to understand if the IBH-PC toolkit is more or less effective for various patient and practice groups. Specific patient subgroups to be tested include gender, age in quartiles, race (white vs. other), education (less than high school graduation, high school diploma, college degree), degree of multi-morbidity (2 vs. > 2 qualifying conditions), and each of the qualifying conditions.

Specific practice subgroups will be analyzed based on practice size, staffing, geography, and financial structure.

These subgroup analyses will follow the same plan as the primary analysis except that an additional characteristic (representing subgroup membership) will be included as well as its interaction with the experimental condition.

### Exploratory analyses

We will replace the binary predictor variable (active vs. control) with continuous variables (the PIP total score and its subscales) to answer the question: What is the relationship between a practice’s degree of behavioral health care integration and patient-centered outcomes?

### Methods in analysis to handle protocol non-adherence and any statistical methods to handle missing data {20c}

We will employ the “intention-to-treat” principle; analyses will be conducted according to a practice’s randomization assignment. The final analysis population will be fully described and differences from the enrolled population will be presented. An advantage of GLM models with time of measurement as an explicit covariate is that equal numbers of data points and equally spaced intervals between data points are not required [68], so subjects without complete data still contribute to the analysis. Cases that are lost may remain in the analysis, although they will provide less information and reduce the variance of estimated effects less than cases that are present for all measurement times.

### Plans to give access to the full protocol, participant level-data and statistical code {31c}

The study is registered on ClinicalTrials.gov (NCT02868983). A complete, cleaned, de-identified copy of the final dataset used in conducting the final analyses will be made available within 1 year of study completion. It will include a data dictionary with response options and missing values defined as well as a complete set of survey instruments (excluding copyright-protected material not licensed for transfer). The data will be available as an Stata data set or comma-separated file. We will not make data from qualitative results available because of the potential for identifying individuals. Despite redacting interviewees’ names from their transcripts, there remains potential for identification by other information shared throughout the interviews. This is particularly relevant given our focus on contextual factors, which necessarily include information about the practice and surrounding community. We concluded that it would not be feasible to determine all of the potentially identifiable data that would need to be redacted to protect the identity of interviewees.

Outside investigators who wish to use the data may request them from the PI. They will be encouraged to collaborate with the research team as appropriate. If the research question they propose is not already under analysis by a member of the research team, the data will be encrypted and sent via SFTP or other secure transfer protocol. If outside investigators wish to reproduce any of our analyses, a full description of our methods (including statistical programming scripts in Stata) as well as consultation by the PI, statistical investigators or others as appropriate, will be made available.

Outside investigators will be required to follow all the protocols for confidentiality, security, notifications to the study sponsor (PCORI), acknowledgments of funding, etc. required of the research team.

### Oversight and monitoring

#### Composition of the coordinating center and trial steering committee {5d}

The research team includes expertise in behavioral and medical management, research design, multicenter data collection, epidemiology and biostatistics, patient engagement, practice transformation, continuing education, quantitative outcomes assessment, and qualitative analysis of processes and outcomes. The team also includes several patients and other stakeholders from the multiple chronic conditions community who provide expertise from lived experience.

#### Composition of the data monitoring committee, its role and reporting structure {21a}

The Data and Safety Monitoring Board (DSMB) is an independent group that advises the IBH-PC study investigators. The members of the DSMB serve in an individual capacity and provide their expertise and recommendations. They report to the PI.

The primary responsibilities of the DSMB are to (1) periodically review and evaluate reports of potential adverse events reported by study participants, personnel, or others; (2) determine the causality and severity of adverse events; and (3) make recommendations for changes in study protocols, operations, or consent procedures as a result of their findings. The DSMB considers study-specific data as well as relevant background knowledge of the patient population under study.

The chair of the DSMB is a patient. Additional members include a PCP, a Psychiatrist, and a BHP.

The DSMB meets at least annually and whenever the PI determines that a significant number of adverse events have been reported or a single adverse event report is potentially serious and warrants DSMB review.

### Adverse event reporting and harms {22}

Although the intervention is low risk, it is possible that a research participant could suffer an unanticipated or adverse event related to the study. If such a concern is raised by study staff, a participant, an investigator, clinician, or any other party, the event is reported to the Institutional Review Board (IRB) and reviewed by the DSMB.

The DSMB determines the causality of each adverse event as “unrelated,” “related,” or “possibly related” to the IBH-PC toolkit or other aspects of the study. It determines the severity of each adverse event according to the Common Terminology Criteria for Adverse Events [69] (CTCAE). Recommendations for changes in the consent process or study protocol may be made, based on the nature of the adverse event.

### Frequency and plans for auditing trial conduct {23}

There are no plans for auditing trial conduct.

### Plans for communicating important protocol amendments to relevant parties (e.g., trial participants, ethical committees) {25}

Changes to the study protocol are communicated to all research team members electronically and reviewed in accordance with IRB policies. Through regular meetings with the study sponsor (PCORI), changes are discussed and tracked through a regular milestone reporting process.

### Dissemination plans {31a}

Dissemination is a standing agenda item for the Investigators and the Stakeholder Advisory Group (SAG). Research team and SAG members, as well as clinicians and patients at the clinical sites, may be asked to make presentations to health care providers, accountable care organizations (ACO), primary care organizations, health-related agencies, legislative committees, and business forums (employer and payer organizations) in their communities. Patient advisors will be asked to review all materials so that findings are understandable and usable by a broad audience. It is anticipated that study findings will be reported in numerous formats such as peer-reviewed literature, conference proceedings, blog posts, and policy briefs.

## Discussion

As IBH gains momentum and primary care practices are encouraged to provide integrated behavioral health services, evidence-based interventions that are shown to effectively increase practice integration levels will be important. This study evaluates the effectiveness of an evidence-based practice change intervention to improve patient outcomes in a vulnerable population and to increase a practice’s degree of integration. There are a number of strengths to our design. First, we are able to overcome a major obstacle in the study of integrated versus co-located practices by randomizing all practices and using co-location as the control condition. Second, we will be able to understand the impact of the IBH-PC toolkit on multiple domains of health and practice integration. Third, our patient recruiting procedure uses a trusted third party to consent patients so practices can remain blind to their patients’ participation status -- an important feature that reduces biases as practices apply newly-learned care approaches with their patients. Finally, the pragmatic nature of the intervention is a strength, as it provides flexibility in how the IBH-PC toolkit is used and which components of integration a practice chooses to apply at any given time.

The pragmatic study design is also a limitation in that it will be difficult to ascertain which aspects of the intervention are associated with any potential improved outcomes. However, supporting flexibility in how and when practices engage with the intervention is important for practice retention, and we will be able to examine which components of the IBH-PC toolkit are most vs. least commonly used by individual practices. Another study limitation is that, due to the complexity of longitudinal EMR extraction across multiple sites, we will not evaluate the impact of the intervention on physiologic outcomes such as blood pressure and hemoglobin A1C.

The study will provide both clinical trial and contextual information that is vital to efforts to improve the integration of behavioral and primary medical care [70, 71].

## Trial status

The IBH-PC study information is available on ClinicalTrials.gov, protocol version 4, April 16, 2019. Practice recruitment began when the study was funded on April 1, 2016. Currently, 45 primary care practices have been recruited for study participation. Two of these practices are participating as “Vanguard” (pilot) sites and are not contributing data to the main analyses. One practice withdrew from the study, leaving 42 participating practices. Twenty of these practices are randomized to the active arm and 22 to the control arm.

Patient participant recruitment began in September 2017. Currently, there are 3006 patient participants who have completed baseline surveys and may be eligible for the main analyses if they complete follow-up surveys. Follow-up data collection concluded in December 2020. Data processing is underway.

Eight IBH-PC active practices have been recruited for inclusion in the qualitative case study analysis focused on examining factors associated with supporting or impeding behavioral health integration and data collection is complete. For the cost assessment analysis, 8 IBH-PC active practices were been recruited and data collection is complete. Recruitment and data collection were completed in December 2020.

Due to the complex nature of the study intervention and potential need to respond with modifications, the study team chose to submit the protocol for publication near the conclusion of the follow-up data collection period in order to ensure accuracy in the reported details.

## Data Availability

We will publish the participant-level, de-identified data used to analyze research questions 1 and 2, in a publicly available online data repository within 12 months of study conclusion. Data used to analyze research questions 3 and 4 will not be publicly available due to concerns with de-identification.

## Abbreviations

ASUI: Asthma Symptom Utility Index
BH: Behavioral Health Care
BHP: Behavioral Health Provider
CAT: Cost Assessment Tool
CI: Confidence interval
CARE: Consultation and Relational Empathy
DSMB: Data and Safety Monitoring Board
DASI: Duke Activity Status Index
EMR: Electronic Medical Record
GLM: Generalized Linear Mixed
GAIN-SS: Global Assessment of Individual Needs - Short Screener
GAD: Generalized Anxiety Disorder Scale
HIPAA: Health Insurance Portability and Accountability Act
IRB: Institutional Review Board
IBH: Integrated Behavioral Health Care
IBH-PC: Integrating Behavioral Health and Primary Care study
IP: Internet Protocol Address
MGLS: Modified Self-reported Medication-taking Scale, Morisky Green Levine Scale
NCQA: National Committee for Quality Assurance
PCI: Patient Centered Index
PCORI: Patient-Centered Outcomes Research Institute
PHQ: Patient Health Questionnaire
PROMIS: Patient-Reported Outcomes Measurement Information System
PIP: Practice Integration Profile
PCP: Primary Care Providers
PI: Principal Investigator
QI: Quality Improvement
REDCap: Research Electronic Data Capture
SRHI-A: Self-Report Habit Index-Alcohol
SAG: Stakeholder Advisory Group
UVM: University of Vermont
